# Autophagy-related *Djatg8* is required for remodeling in planarian *Dugesia japonica*

**DOI:** 10.1242/bio.045013

**Published:** 2019-12-03

**Authors:** Jing Kang, Zimei Dong, Jing Wang, Guangwen Chen, Dezeng Liu

**Affiliations:** 1 College of Life Science, Henan Normal University, Xinxiang 453007, China; 2 College of Life Science, Xinxiang Medical University, Xinxiang 453003, China

**Keywords:** Planarian, Autophagy, *Djatg8*, Regeneration, Remodeling

## Abstract

Planarians are the earliest free-living platyhelminthe with triploblastic and bilateral-symmetry. As an integral component of tissue homeostasis and regeneration, remodeling occurs constantly in the general planarian life history. In the present study, we isolate three planarian *Dugesia japonica Atg8* genes (*Djatg8-1*, *Djatg8-2*, *Djatg8-3*) that show high sequence similarity with *Atg8* from yeast and human. Results from whole-mount *in situ* hybridization indicate that *Djatg8-2* and *Djatg8-3* are strongly expressed in blastemas during *Dugesia japonica* regeneration. Using RNA interference, inhibition of *Djatg8-1* gene expression has no obvious effect on planarian morphological changes. Interestingly, downregulation of *Djatg8-2* gene expression in planarians results in defects in blastema regeneration and tissue regression. Furthermore, loss of *Djatg8-3* expression leads to tissue degradation. Taken together, our results suggest that *Djatg8-2* and *Djatg8-3* play important roles in planarian remodeling during regeneration.

## Abstract (precis)

**Summary:** The autophagy-related *Djatg8* homologues *Djatg8-2* and *Djatg8-3* are required for planarian remodeling; we provide an emergent *in vivo* model organism to study autophagy and the molecular mechanism for planarian remodeling.

## INTRODUCTION

Planarians are well known to have a powerful regenerative ability and are considered similar to ancestral animals (Dong et al., 2015). Planarians have been recognized as a classic model to study tissue regeneration due to their amazing characteristics (Saló, 2006; González-Estévez, 2009). During regeneration, planarians undergo continuous body remodeling and no food can be taken until the formation of a new functional pharynx (Ma et al., 2018a). The question in regeneration is how exactly the energy is supplied. Autophagy is a physiological process of energy and protein turnover that can be rapidly upregulated for supplying adenosine triphosphate (ATP) and amino acids from the degradation products of lysosomes when the organism is undergoing architectural remodeling (Mizushima and Komatsu, 2017; Toshima et al., 2014). Therefore, it has been proposed that autophagy plays an essential role in regeneration (González-Estévez, 2008). In contrast to to other models, planarians can provide an *in vivo* model and may be used to study autophagy (González-Estévez, 2009).

Autophagy is a lysosome-mediated process in which the cytoplasmic cargos, including aged proteins, misfolded proteins or damaged organelles, are sequestered in double- or multi-membrane vesicles called autophagosomes and are delivered to lysosomes for bulk degradation. In recent years, a series of *Atg* genes have been shown to be involved in autophagic processes (Shao et al., 2016; Krick and Thumm, 2016). Among them, autophagy-related protein 8 (Atg8), a lipid-conjugated ubiquitin-like protein, plays an important role in the formation of autophagosomes, membrane extension and the identification of specific substances. Atg8-PE (phosphatidylethanolamine, PE) is one of the ubiquitin-like conjugation proteins and is required for the autophagosome formation (Wang et al., 2015; Kalvari et al., 2014; Krick and Thumm, 2016). Atg8 initially localizes in the phagophore assembly site (PAS). During maturation of the autophagosome, the Atg8 protein is trapped inside and eventually degraded (Kalvari et al., 2014; Krick and Thumm, 2016).

*Atg* genes are essential regulators of regeneration and development, and are involved in the regulation of autophagy function. In *Drosophila*, increasing *Atg1* expression can elevate autophagy and suppresses tissue degeneration by promoting mitochondrial fission (Ma et al., 2018b). In *Caenorhabditis elegans*, the *Atg8* homolog (*lgg-1*) is essential for embyro development, and *atg-3* and *atg-7* are required for LGG-1 conjugation to PE, which plays a key role in autophagy (Zhang et al., 2015). However, more than 10 years have lapsed since Gonzalez-Estevez (González-Estévez, 2008, 2009) proposed that planarians could serve as a new model organism for studies on autophagy and limited research on autophagy has been performed at the molecular level (Ma et al., 2018a). It has not been shown that *Atg* in planarians can affect planarian regeneration. In this work, we focus on the characterization of *Atg8* during planarian regeneration and body remodeling.

## RESULTS

### Cloning of *Djatg8* genes

**Fig. 1.**
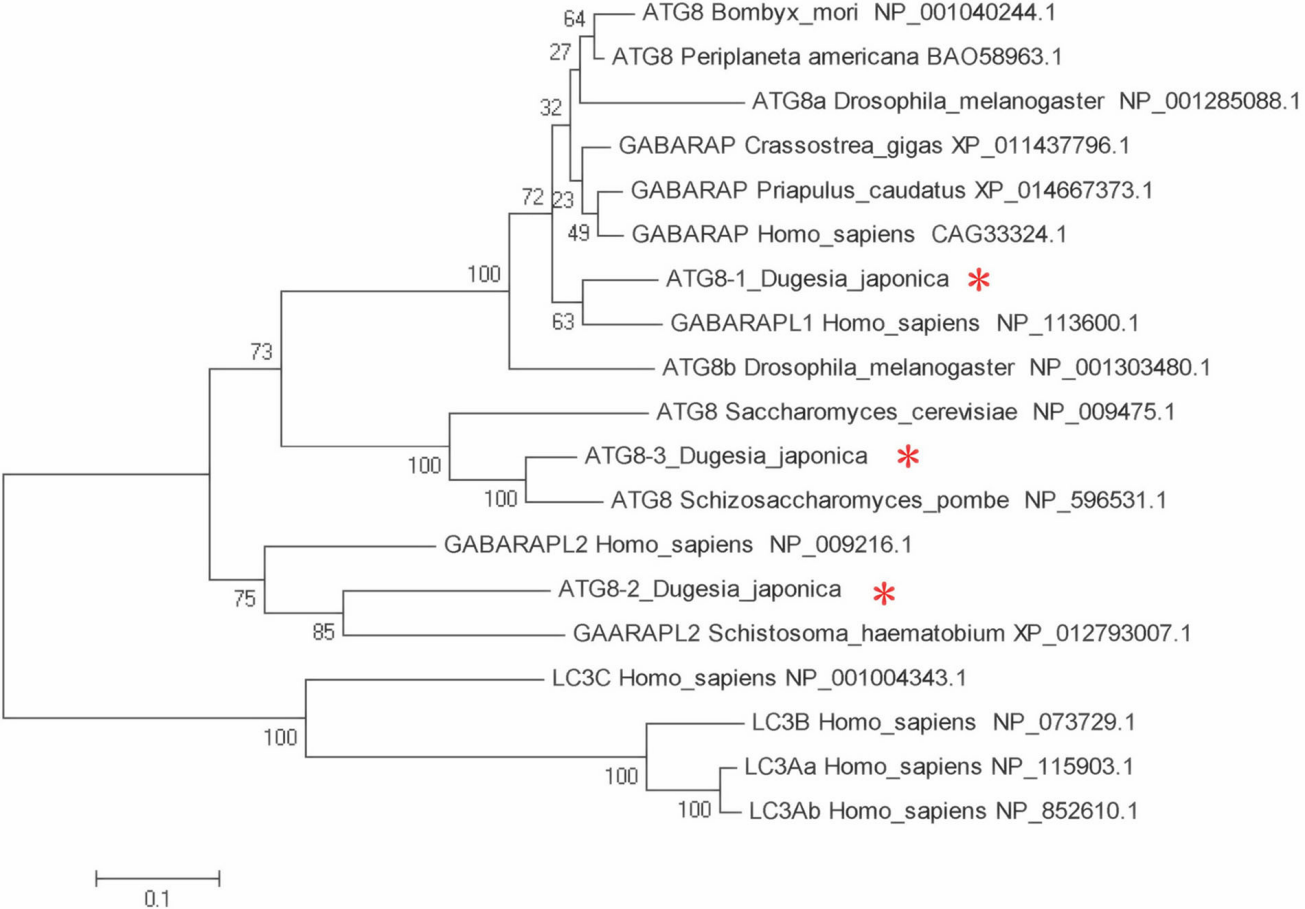
**Phylogenetic analysis of *Djatg8* sequences.** The tree was constructed by the neighbor-joining method. *Djatg8* marked by asterisks. The bootstrap confidence values were calculated from 1000 replications.

From yeast to mammals, autophagy is an important mechanism for sustaining cellular homeostasis through facilitating the degradation and recycling of aged and cytotoxic components (Yoshimori and Noda, 2008; Kourtis and Tavernarakis, 2009; Xie et al., 2008; Cárdenas-Zuñiga et al., 2016). In yeast, autophagy initiation, cargo recognition, cargo engulfment and vesicle closure is Atg8-dependent (Yoshimori and Noda, 2008; Kourtis and Tavernarakis, 2009; Xie et al., 2008; Cárdenas-Zuñiga et al., 2016). In mammals, Atg8 belongs to the LC3/GABARAP protein family, which consists of seven family proteins [LC3A (two splice variants), LC3B, LC3C, GABARAP, GABARAPL1 and GABARAPL2] (Marco et al., 2016). LC3B, the well-investigated family protein, is associated with autophagosome development and maturation and is used to monitor autophagic activity (Marco et al., 2016). In planarian *D. japonica*, three *Atg8* orthologs were identified and named *DjAtg8-1*, *Djatg8-2* and *Djatg8-3* (GenBank accession numbers: KY050772, KY050771 and KY050773). The full-length *Djatg8-1* encodes 117 amino acids, the full-length *Djatg8-2* encodes 119 amino acids and the full-length *Djatg8-3* encodes 118 amino acids. NCBI blast shows that the Djatg8-1 amino acid sequence is 61.3% identical to *Homo sapiens* GABARAPL1, and the Djatg8-2 amino acid sequence is 71% identical to *Schistosoma haematobium* GABARAPL2*.* The Djatg8-3 amino acid sequence is identical to two yeast Atg8 sequences: 95% in *Saccharomyces haematobium* and 92% in *Saccharomyces cerevisiae*. The phylogenetic tree (Fig. 1) shows that the planarian Djatg8-3 and two yeast Atg8 are clustered with a boot strap percentage of 100%, the planarian Djatg8-2 and *S. haematobium* GABARAPL2 are clustered with a boot strap percentage of 85% and the planarian Djatg8-1 and *Homo sapiens* GABARAPL1 are clustered with a boot strap percentage of 63%.

### *Dugesia japonica* possesses a putative Atg8 protein

**Fig. 2.**
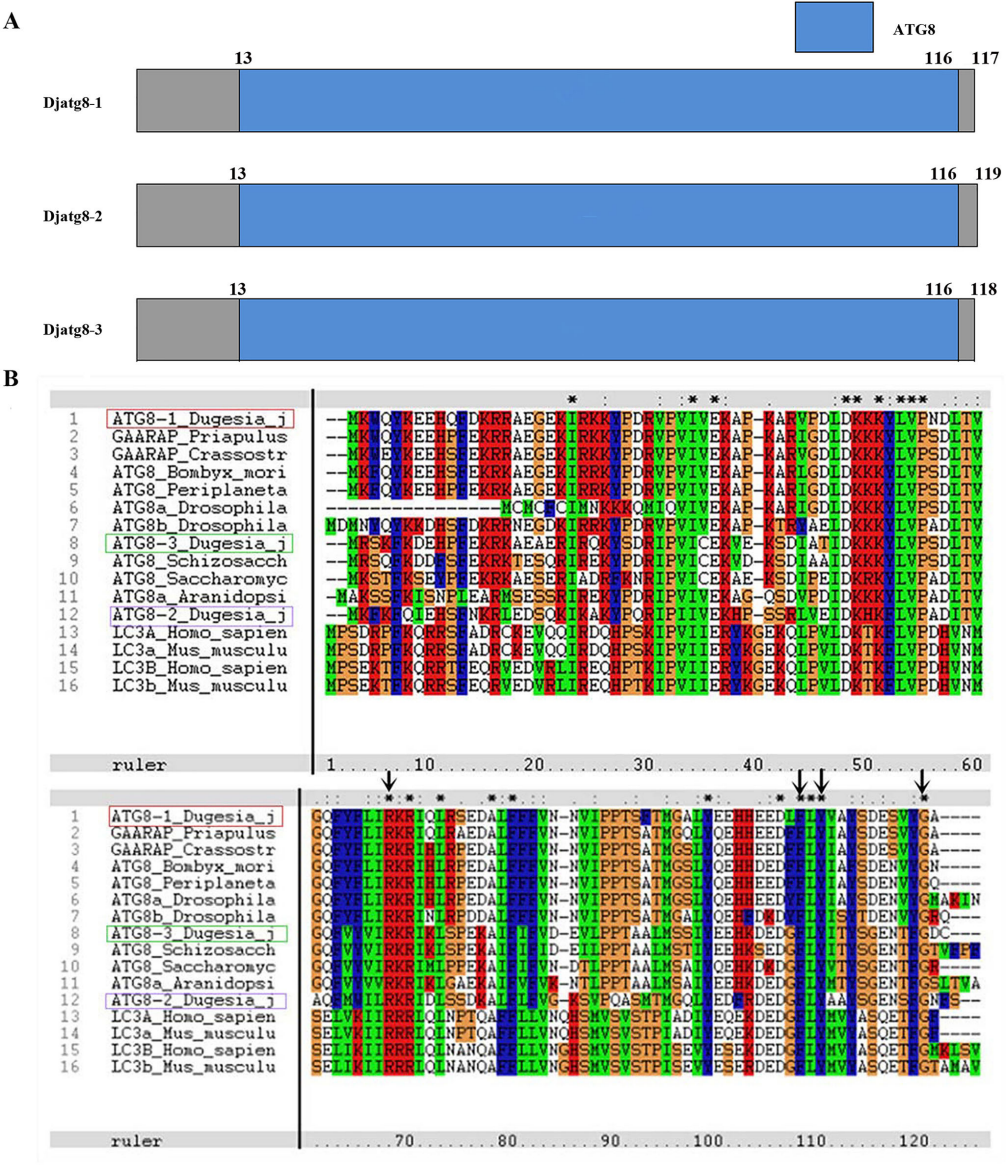
**Djatg8-conserved domains.** (A) Schematic illustration of the domains present in Djatg8s. (B) Amino acid sequence alignment of Atg8 family members. Arrows point out Arg65, Phe104, Tyr106 and C-terminal glycine; *, highly homologous.

**Fig. 3.**
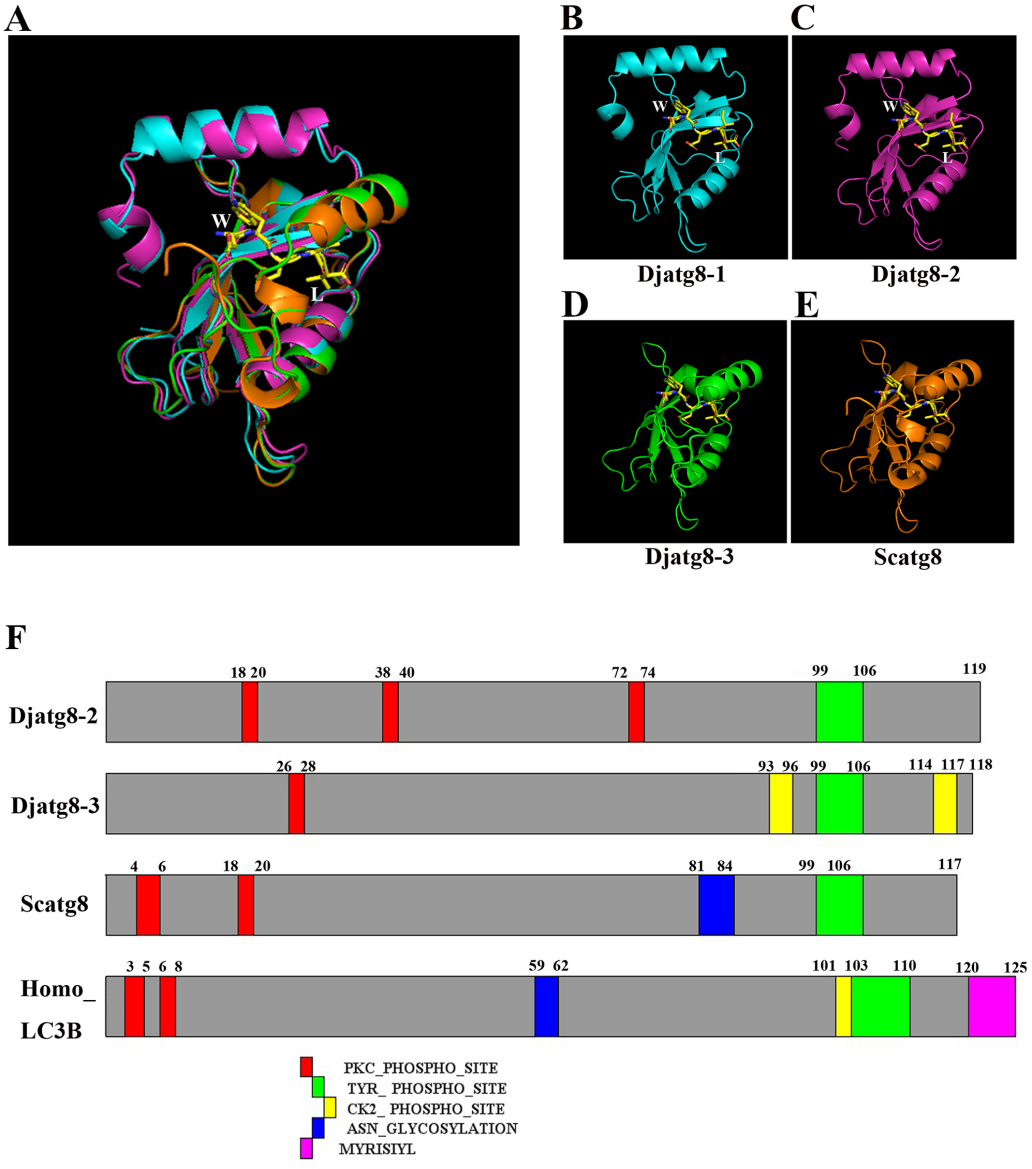
**Phosphorylation site predictions and structures of Atg8s.** (A–E) Structures of Djatg8s and AIM. Peptide is shown in yellow. (F) Phosphorylation site predictions of Atg8 proteins. Djatg8-2 and Djatg8-3 from *D.* *japonica*, Scatg8 from *Saccharomyces cerevisiae*, LC3B from *Homo sapiens* (red box, protein kinase C phosphorylation sites; green box, tyrosine kinase phosphorylation sites; yellow box, casein kinase II phosphorylation site; blue box, n-glycosylation site; pink box, n-myristoylation site).

A recent study has shown that Arg65, phe104 and Tyr106 in yeast ATG8 are highly conserved residues that are essential for the conjugation of ATG8 to PE and the C-terminal glycine (Ling et al., 2015). Adjacent amino acids of yeast ATG8 have been identified as essential residues that are cleaved by ATG4 to expose the glycine residue before conjugating to PE, which is catalyzed by ATG7 and ATG3 (Ling et al., 2015). To examine whether *D. japonica* possesses an Atg8 homolog, we performed multiple alignment analysis with atg8 sequences. Our results reveal that *Djatg8* contains these three conserved residues at the corresponding positions (Fig. 2B). These results indicate that three polypeptides in *D. japonica* (*DjAtg8*) correspond to an Atg8 homolog protein. SMART data show that a putative Djatg8 displays the conserved ATG8 domains (Fig. 2A). The Djatg8 tertiary structures, in a complex with a peptide containing Atg8 interaction motif (AIM), reveal the similar domain organizations seen in ATG8 proteins from mammals and yeast (Fig. 3A–E). The AIM peptide was buried in two distinct pockets (W and L) of ATG8 (Fig. 3A–E), and Djatg8-3 has similar structures in the yeast ATG8 protein (Fig. 3D,E). The ‘W’ pocket is found in the region between the N-terminal/-helices and the b-grasp, and embraces the aromatic residue of AIM; The ‘L’ pocket is located within the ubiquitin-like fold and embraces the branched-chain amino acid in the core AIM (Kellner et al., 2016). The conserved phosphorylation site analyses showed that Djatg8-2 and Djatg8-3 proteins contain four phosphorylation sites that are similar to yeast and human Atg8, but the Djatg8-1 protein has no PROSITE signature (Fig. 3F). Our results reveal that Djatg8 belongs to the Atg8 family; Djatg8-3 has a high similarity with Atg8 from yeast in the structure.

### *Djatg8-2* and *Djatg8-3* are upregulated during planarian regeneration

**Fig. 4.**
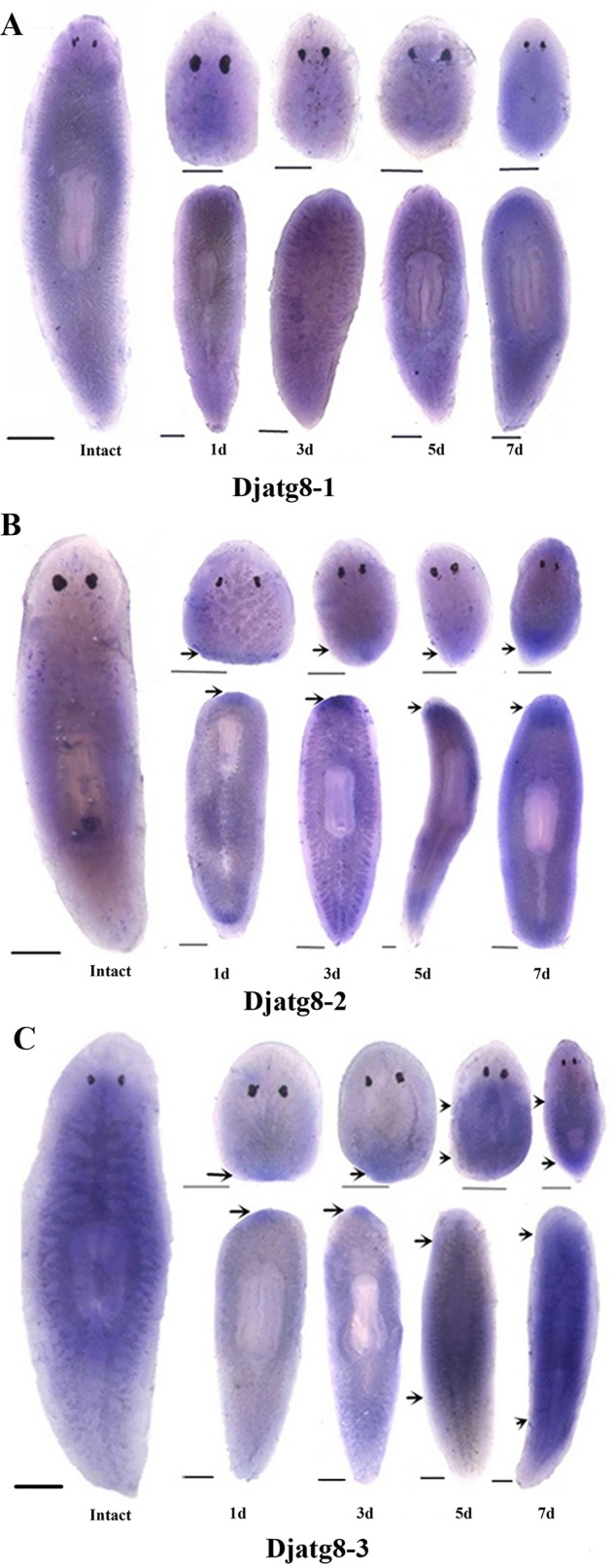
**WISH in intact and regenerating planarians.** (A) WISH of *Djatg8-1* in intact and regenerating planarians. (B) WISH of *Djatg8-2* in intact and regenerating planarians. (C) WISH of *Djatg8-3* in intact and regenerating planarians. Arrows show the regions where expressions are upregulated. d, day. *n*=12 animals. Scale bars: 500 μm.

Next, the expression patterns of *Djatg8* were analyzed after amputation in planarians. Regeneration can be induced by one amputation just anterior to the pharynx and posterior to the auricle, producing two pieces (head and tail pieces), which can regenerate missing parts within 1 week at 20°C. Whole-mount *in situ* hybridization (WISH) (Fig. 4) and RT-PCR (Fig. S1) results show that *Djatg8-1* mRNA expression has no significant difference in regenerated worms (Fig. 4A; Fig. S1A). *Djatg8-2* (Fig. 4B) and *Djatg8-3* (Fig. 4C) were strongly expressed in both sides of the body and poorly expressed in the pharynx in intact worms. During regeneration, *Djatg8-2* mRNA expression was detected and gradually increased from 1 to 7 days in blastemal cells (Fig. 4B; Fig. S1B). At 1 and 3 days, *Djatg8-3* was highly expressed in the post-blastemas, which surrounded the wound (Fig. 4C). The expression of *Djatg8-3* gradually increased after 3 days of regeneration (Fig. 4C; Fig. S1C) and higher levels were found throughout the parenchyma and the digestive systems of 5- and 7-day-regenerated pieces (Fig. 4C). Those results coincide with the time of the formation of the new body of the planarian (Fig. 4; Fig. S1).

### *Djatg8-2* RNAi induce disproportionate blastemas with tissue regression during regeneration

To investigate the roles of Djatg8 proteins during planarian regeneration, we performed RNAi-knockdown experiments. The effectiveness of RNAi was detected by WISH (Fig. S2A) and RT-PCR (Fig. S2B). The results revealed that endogenous *DjAtg8* expression was significantly lower in RNAi animals than in control animals (Fig. S2). The process of planarian regeneration is special, since following amputation or injury, the planarian can completely regenerate all missing structures in about 7 days. Because no abnormal phenotypes were observed after *Djatg8-1* RNAi (Fig. S3), we focused on *Djatg8-2* and *Djatg8-3*.

**Fig. 5.**
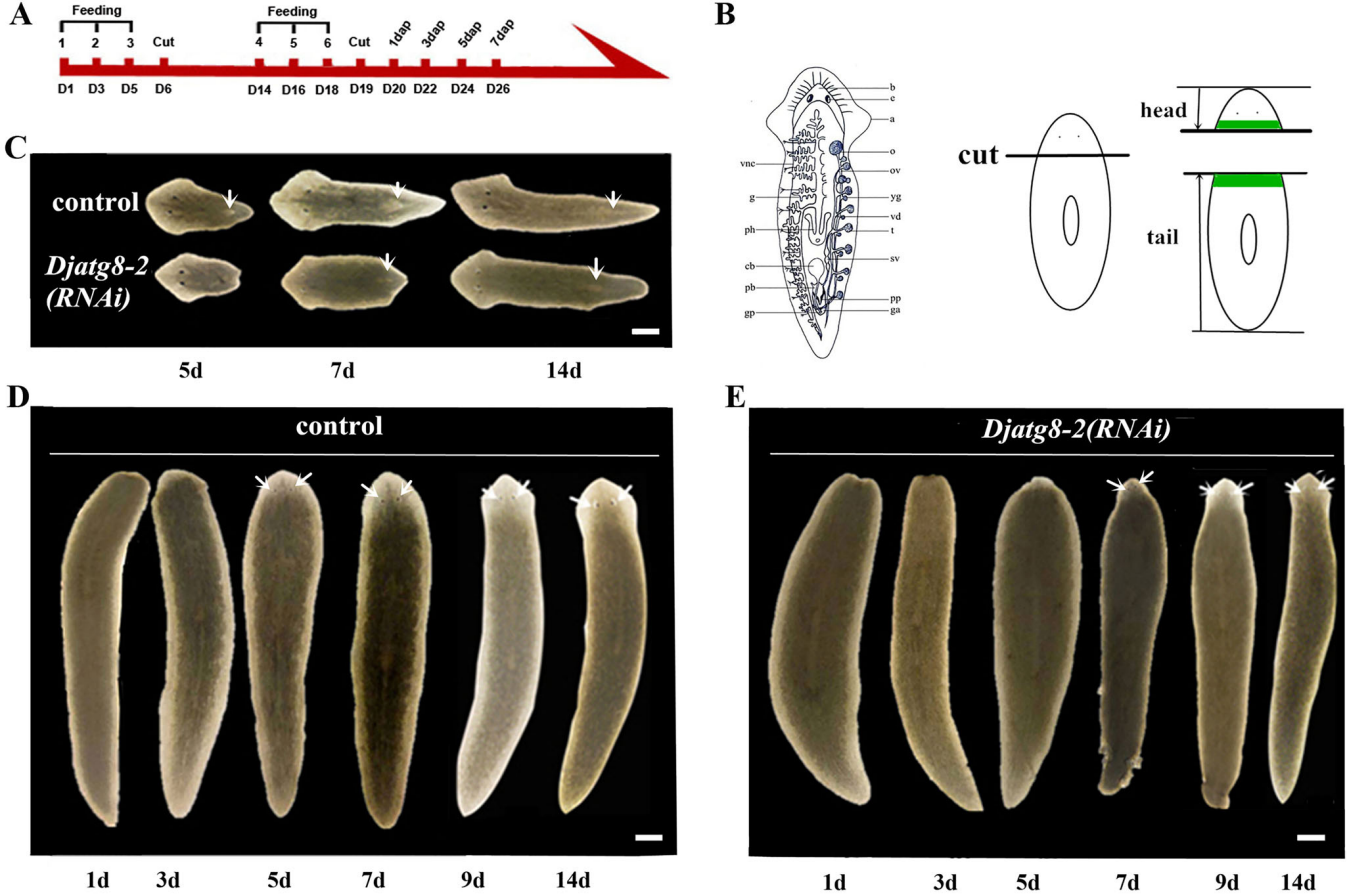
**Effects of *Djatg8-2* RNAi treatment in planarians.** (A) Flowchart of dsRNA feeding and amputation schedules (dap, days after second amputation; D, days after the first dsRNA feeding). (B) Schematic illustrations of sexually mature *D. japonica* (vnc, ventral nerve cords; g, gut; ph, pharynx; cb, copulatory bursa; pb, penis bulb; gp, genital pore; b, brain; e, eye; a, auricle; o, ovary; ov, oviduct; yg, yolk gland; vd, vas deferens; t, testis; sv, seminal vesicle; pp, penis papilla; ga, genital atrium; ca, common atrium; Dong et al., 2015) and of amputation, where green areas show blastemas. (C) Head regeneration at 5, 7 and 14 days after *Djatg8-2* RNAi-treatment (white arrows, pharynx). (D) Tail regeneration at 1, 3, 5, 7, 9 and 14 days in controls (white arrows, eyes). (F) Tail regeneration at 1, 3, 5, 7, 9 and 14 days with disproportionate blastemas and eyes (white arrows) after *Djatg8-2* RNAi-treatment. *n*=20 animals for each treatment. Scale bars: 500 μm.

Control planarians can restore all missing structures in about 7 days and need only 7 more days to completely rescale to their new size (González-Estévez and Saló, 2010). All control (RNAi) (*n*=20/20) planarians fully regenerated and displayed no apparent phenotype during regeneration (Fig. 5C,D). Worms treated with *Djatg8-2* RNAi displayed slower regeneration speed compared with controls (Fig. 5C–E). In head fragments, the majority of *Djatg8-2* (RNAi) planarians showed profound tissue regression with the new pharynx not observed on the fifth day of regeneration (Fig. 5C). In addition, the regenerated head pieces formed smaller tails and pharynxes than controls on the seventh day of regeneration (Fig. 5C), while the regenerated tail pieces formed smaller heads than controls on the seventh and ninth days (Fig. 5E) of regeneration. The regenerated tail pieces also showed notable tissue regression, with smaller blastemas from 1 day of regeneration and disproportionate heads at 7 and 9 days (Fig. 5E). Moreover, *Djatg8-2* RNAi induced disproportionate blastemas with tissue regression during regeneration.

### *Djatg8-3* RNAi induces death during regeneration

**Fig. 6.**
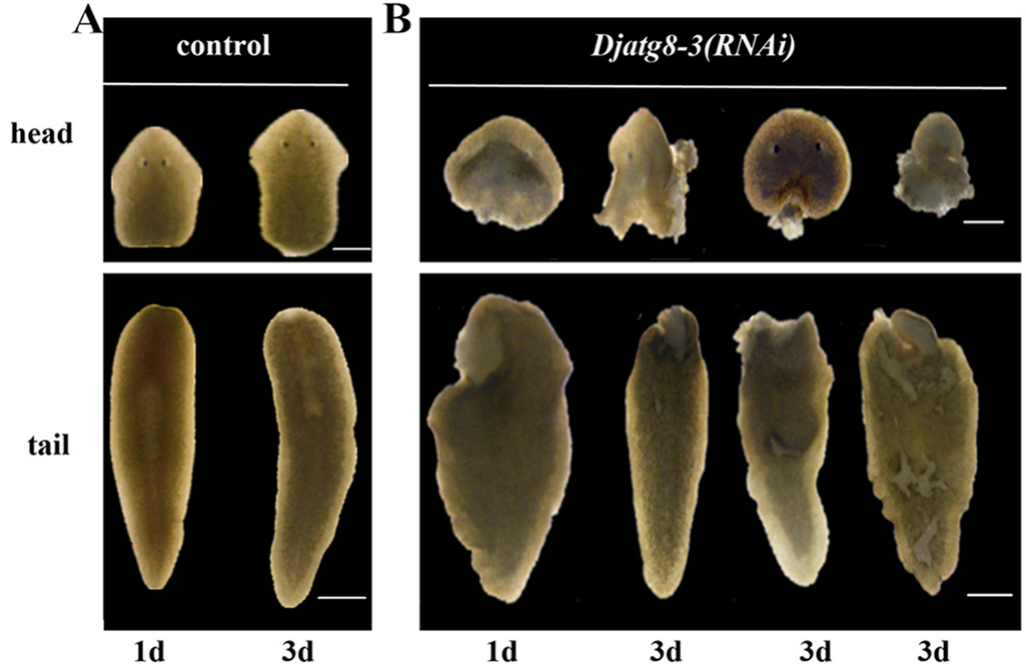
**Planarian phenotypes after *Djatg8-3* RNAi treatment (*n*=20 animals for each treatment).** (A) Head and tail after 1 and 3 days of regeneration in control. (B) Head and tail after 1 and 3 days of regeneration with lesions in Djatg8-3 RNAi worms. Scale bars: 500 μm.

A significant degradation in regeneration speed was found for *Djatg8-3* RNAi planarians during regeneration, and this degradation was often observed alongside the formation of blastemas. Head and tail pieces underwent lysis on the first day of regeneration, which was followed by death after a few days (Fig. 6). The remaining pieces showed the same phenomenon on the third day of regeneration (Fig. 6) and all *Djatg8-3* RNAi planarians died at 5 days. We interpreted these phenotypes to reflect a failure or reduction in regeneration speed along with remodeling deficiencies in the older parts of the body.

### *Djatg8-3* RNAi induces karyolysis in the nucleus of planarian

**Fig. 7.**
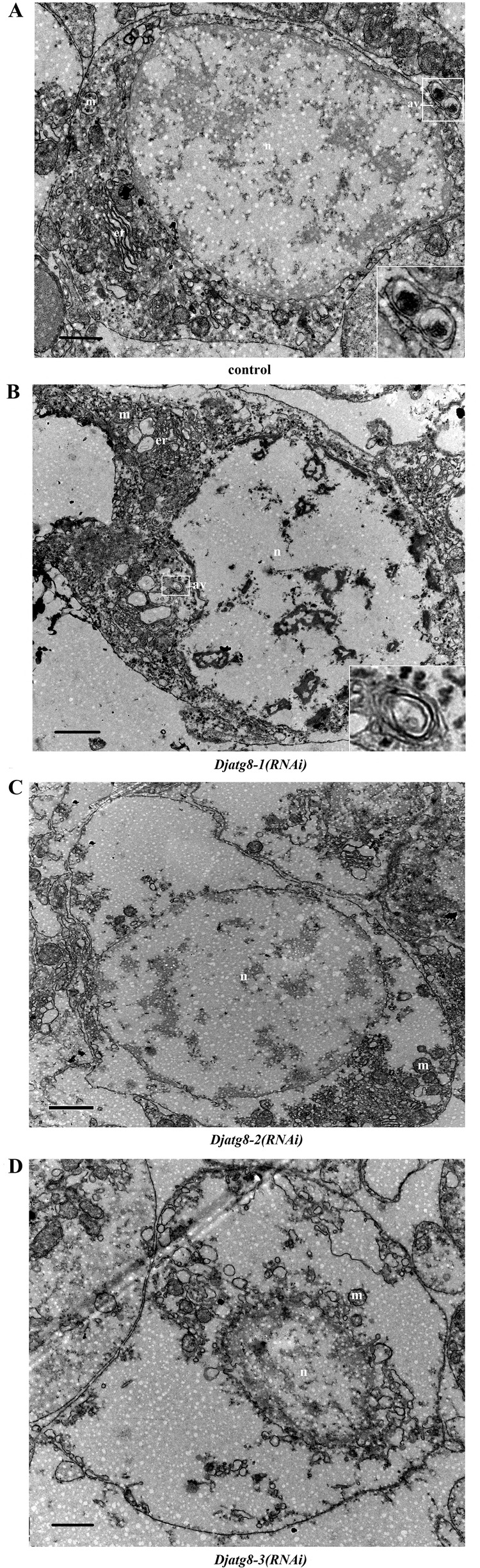
**Ultrastructural changes in** ***D. japonica* induced by *Djatg8* RNAi (*n*=3 animals for each treatment).** (A) Ultrastructural changes in control planarians (av is magnified in the inset). (B) Ultrastructural changes in *Djatg8-1* RNAi planarians (av is magnified in the inset). (C) Ultrastructural changes in *Djatg8-2* RNAi planarians. (D) Ultrastructural changes in *Djatg8-3* RNAi planarians. n, nucleus; m, mitochondrion; er, endoplasmic reticulum; av, autophagic vacuoles. Scale bars: 1 μm.

To show the role of *Djatg8* during tissue turnover, transmission electron microscopy (TEM) was used to analyze ultrastructural changes after treatment with *Djatg8* RNAi. The autophagic vesicles were observed in *Djatg8-1* RNAi and control planarians (Fig. 7A,B), but not in *Djatg8-2* and *Djatg8-3* RNAi animals (Fig. 7C,D). At the same time, some endoplasmic reticulums were observed in control (Fig. 7A) and *Djatg8-1* RNAi worms (Fig. 7B). Furthermore, karyolysis was found in the nucleus in *Djatg8-3* RNAi worms (Fig. 7D).

**Fig. 8.**
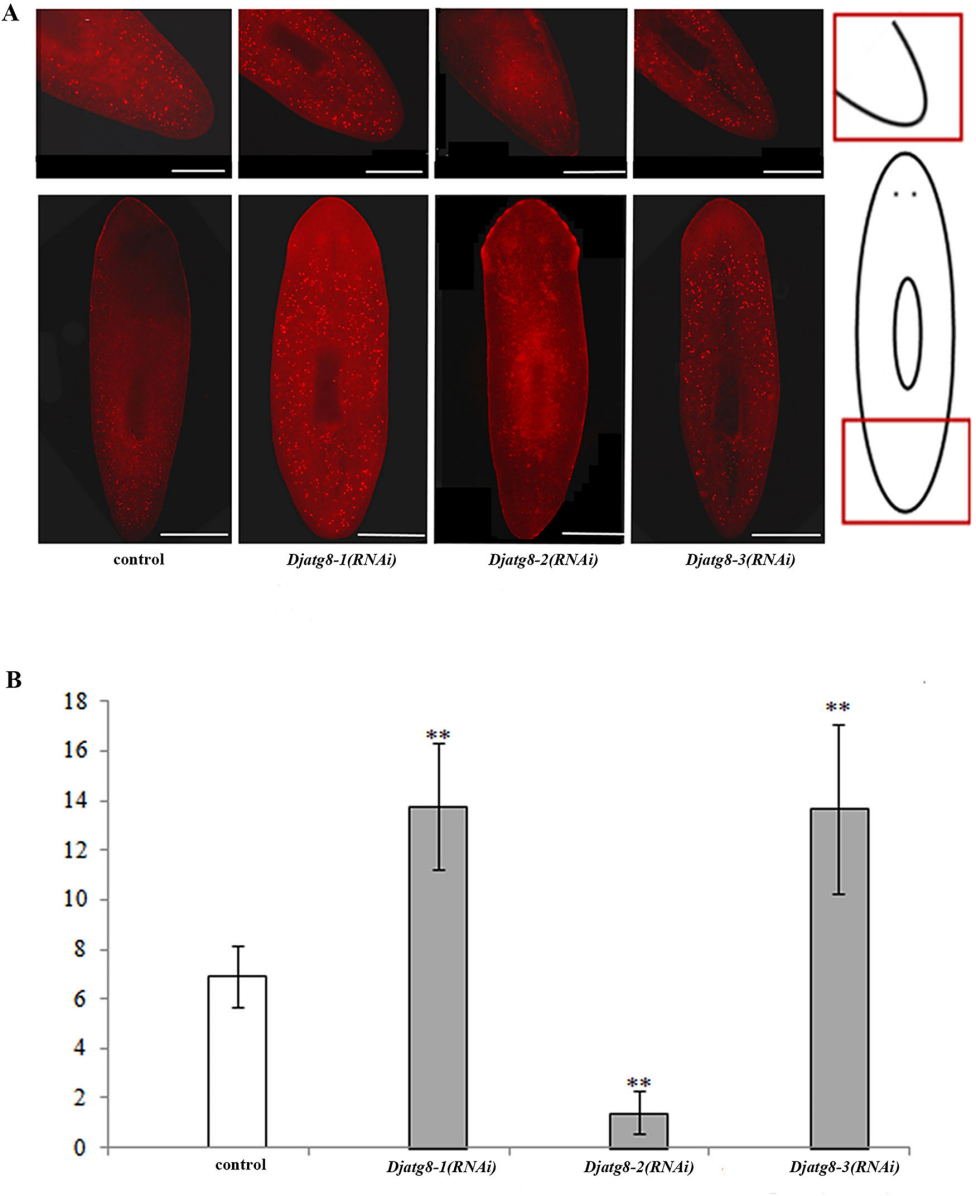
**Whole-mount immunostaining following RNAi treatment in intact worms (*n*=20 animals for each treatment).** (A) Immunostaining with an anti-H3P antibody following *Djatg8-1*, *Djatg8-2* and *Djatg8-3* RNAi treatment (diagrams indicate section of worm magnified). Scale bars: 500 μm. (B) Mitotic density in intact planarian after RNAi treatment (**P<0.01).

To analyze the relation between cell proliferation and gene inhibition in tissue turnover, we performed immunostaining using H3P antibody (González-Estévez et al., 2007a). Rises in mitotic activity were observed in *Djatg8-1* and *Djatg8-3* dsRNA-fed planarians and reductions were seen in *Djatg8-2* dsRNA-fed planarians during tissue homeostasis (Fig. 8).

## DISCUSSION

Dysregulation of autophagy leads to serious disorders in humans (Gangloff et al., 2004). In the present study, we used planarian autophagy as a model to explore *de novo* mechanisms of regeneration. We isolated three planarian *D. japonica Atg8* genes (*Djatg8-1*, *Djatg8-2*, *Djatg8-3*) that show high sequence similarity with Atg8 from yeast and human. Our results show that *Djatg8-2* and *Djatg8-3* are required for planarian remodeling during regeneration, but *Djatg8-1* is not.

### Autophagy is involved in planarian regeneration

Autophagy is an evolutionary conserved mechanism that occurs continuously to maintain the homeostasis of all tissues (González-Estévez and Saló, 2010). During planarian regeneration, they undergo continuous body remodeling and no food can be taken until the formation of a new functional pharynx (Ma et al., 2018a). Therefore, cell death caused by autophagy is necessary as a natural consequence of restricted food availability to maintain tissue homeostasis (González-Estévez, 2008, 2009; González-Estévez and Saló, 2010; Reddien, 2018). Generally, autophagy is a lysosome-mediated degradation process, and it is a physiological process of energy and protein turnover that can supply ATP and amino acids from degradation products of lysosomes when the planarian is undergoing architectural remodeling (Mizushima and Komatsu, 2017; Toshima et al., 2014). Thus, cell death caused by autophagy would be essential to the old or damaged tissues or organs in adult tissue turnover and regeneration.

Recent reports have shown that autophagy occurs in newly regenerated planarians on days 7 and 10 after amputation through TEM investigation (Ma et al., 2018a). Interestingly, the remodeling process can occur by autophagy activation in *Polycelis tenuis* and only two peaks for acid phosphatase activity (autophagy marker) were observed (Bowen et al., 1982). The first peak was observed at 0–12 h after amputation and was higher than the second (40–48 h post amputation) (Bowen et al., 1982). In *Dugesia dorotocephala* (Coward et al., 1974), acid phosphatase activity peaks were detected at their maximum after 12 h and at minimal or basal activity after 36 h. Furthermore, in *Girardi atigrina*, microscopy showed a differentiated cell undergoing autophagy at the post-blastema level after 5 days regeneration (González-Estévez et al., 2007a,b). These studies suggest that autophagy is involved in planarian regeneration. Using TEM, the autophagic vesicles were observed in *Djatg8-1 RNAi* and control planarians, but not in *Djatg8-2* and *Djatg8-3 RNAi* animals. We theorize that loss of *Djatg8-2* and *Djatg8-3* inhibits the autophagic vesicles forming. In mammals, autophagy activity is usually assessed by western blot against LC3 and TEM. Therefore, we used the conventional LC3 antibody [Cell Signaling Technology (CST), no. 4108] to detect the ratio of LC3-II/LC3-I; unfortunately, no signal was detected (data not shown). We have recently been in touch with manufacturers to ask them to produce the antibodies specific to planarians used in autophagic activity assays to aid future studies. We hope this work will be helpful to autophagy research in planarian.

### *Djatg8-2* and *Djatg8-3* are required for planarian remodeling during regeneration, but not *Djatg8-1*

Autophagy was first found in yeasts wherein more than 30 autophagy-related genes have been identified (*Atg1–Atg32*). The majority of these *Atg* genes are also found in higher eukaryotes, including mammals (Yoshimori and Noda, 2008; Kourtis and Tavernarakis, 2009; Xie et al., 2008; Cárdenas-Zuñiga et al., 2016). The Atg8 protein has been explored as one of the most important Atg molecules in autophagy; it is a ubiquitin-like protein involved in the labeling of autophagic vesicles (AVs), and actively controls their formations (Cárdenas-Zuñiga et al., 2016; Krick and Thumm, 2016). In *D.* *japonica*, three *Atg8* orthologs of higher eukaryotes have been identified, including *Djatg8-1*, *Djatg8-2* and *Djatg8-3*. In this study, WISH results showed that *Djatg8-3* was more strongly expressed in blastemas than *Djatg8-2* at 5 and 7 days of regeneration, while *Djatg8-1* was not strongly expressed during regeneration. Our results reveal that animals treated with *Djatg8-2* RNAi show disproportionate tissue regeneration, while *Djatg8-1* RNAi-treated animals display no obvious morphological changes during regeneration. On the other hand, when *Djatg8-3* RNAi worms were amputated, death followed after a few days. Therefore, we suspect that the *Djatg8-2* and *Djatg8-3* play more important roles than *Djatg8-1* during regeneration of planarians.

In mammals, Atg8 consists of seven family proteins that are different structures and LC3B is a marker protein for detecting autophagy activity (Marco et al., 2016). In *C. elegans*, *Atg8* has two homologs, *lgg-1* and *lgg-2*. *lgg-1* is essential for embryo development and *lgg-2* is not (Zhang et al., 2015). Fan et al. (2015) found that the protein’s structure affected its function after investigating structure/function relationships in LGG-1 and LGG-2. In our study, we found that the three Djatg8 proteins have different amino acid sequences. Moreover, Djatg8-2 and Djatg8-3 proteins contain four phosphorylation sites, but Djatg8-1 has no PROSITE signature. We supposed that the difference in protein structure led to the difference in function, because the protein structure (amino acid sequence or phosphorylation site) is an important part of post-translational modifications of proteins, and is also necessary for several biological activities (Suparji et al., 2016; Han et al., 2017).

In this work, the homolog *Djatg8* was successfully cloned and characterized in *D.* *japonica* for the first time, and the associated roles in regenerative worms were analyzed using WISH and RNAi experiments. Our results reveal that *Djatg8-2* and *Djatg8-3* are required for planarian regeneration and tissue homeostasis. In summary, our work provides important evidence of the role of the *Atg8* gene in planarian regeneration and body remodeling.

## MATERIALS AND METHODS

### Animals

Planarians (*D. japonica*) used in this study were collected from Shilaogong, Hebi City, China. Planarians were fed at 20°C in the laboratory until sexual maturity. Planarians were used in our experiments after starvation for 7 days (Dong et al., 2017).

### TEM

Regeneration was induced by one amputation just anterior to the pharynx and posterior to the auricle and produced two pieces (head and tail pieces) (Fig. 5B), which regenerated missing parts within 1 week at 20°C. For the regeneration of fragments, the blastema cells of the two pieces were observed by TEM as previously described (Harrath et al., 2014).

### RNA isolation and cDNA cloning

Total RNA was extracted with TRIzol reagent (Takara, Dalian, China), and first-strand cDNA was synthesized from 1 μg of total RNA with SuperScript III Reverse Transcriptase (Invitrogen, CA, USA) according to the manufacturer's instructions (Dong et al., 2017). The partial-length cDNA *Djatg8* genes were obtained from the transcriptome provided by the Novogene Company (Bejing, China). Because a *Djatg8-1* cDNA fragment has full open reading frames (ORF), they were deposited into the GenBank database. *Djatg8-2* and *Djatg8-3* full-length transcripts were amplified by rapid amplification of complementary DNA (cDNA) ends using both the 5′- and 3′-Full RACE kits (Takara, Dalian, China) according to the manufacturer's instructions (Dong et al., 2017) . The following sequence-specific primers were used:

Djatg8-2 3′RaceF: 5′-CGCTGCATACAGTGGCGAGAAT-3′,

Djatg8-2 3′RaceR: 5′-CCTCAAGCAAGTATGACGATGG-3′,

Djatg8-2 5′RaceF: 5′-CGGTATTCTTTGCGGATATTTAGC-3′,

Djatg8-2 5′RaceR: 5′-CTGATGATAAATCAATTCGCTTCC-3′,

Djatg8-3 3′RaceF: 5′-AAGTTGTCCCCCGAGAAGGC-3′,

Djatg8-3 3′RaceR: 5′-TCTACGTTATCCGCAAGCGTATC-3′,

Djatg8-3 5′RaceF: 5′-ACGGGAATGCGGTCAGAGT-3′ and

Djatg8-3 5′RaceR: 5′-TTGATACGCTTGCGGATAACG-3′.

### Sequence analysis and phylogenetic tree construction

Similarity analysis of nucleotide and protein sequences was carried out with BLASTn and BLASTp provided by the National Center of Biotechnology Information (http://www.ncbi.nlm.nih.gov/blast) (Dong et al., 2015). Multiple protein sequences were aligned using the Clustalx software. The phosphorylation sites of proteins were predicted by the ExPASy (http://web.expasy.org/prosite/) and SMART (http://smart.embl-heidelberg.de/) programs. Phylogenetic tree analysis was performed on amino acid sequence alignments by the neighbor-joining method using the Mega 5.0 program (Dong et al., 2015). Statistical support was provided by 1000 bootstrap replications.

### WISH

Digoxigenin-labeled RNA probes were prepared with an *in vitro* labeling kit (Roche, Basel, Switzerland) (Dong et al., 2017). Then, WISH was carried out as previously described and developed with NBT/BCIP (Dong et al., 2017). The following sequence-specific primers were used:

Djatg8-1 WISH F: 5′-TCTAGAATGAAGTGGCAGTACAAAGA-3′,

Djatg8-1 WISH R: 5′-AAGCTTTGATGAAAGTGTATATGGCGCA-3′,

Djatg8-2 WISH F: 5′-TCTAGAATGAAATTCAAATTTCAGATTGAA-3′,

Djatg8-2 WISH R: 5′-AAGCTTGGCGAGAATTCCTTTGGCAACTTT-3′,

Djatg8-3 WISH F: 5′-TCTAGAAATCAAATCCACTGTCATTCAT-3′ and

Djatg8-3 WISH R: 5′-AAGCTTCAGGTCCGACGGGACAAGGTA-3′.

### RNAi

Worms were fed bacteria induced to express double-strand RNA (dsRNA) against *Djatg8* genes as previously described (Dong et al., 2017). The bacteria have the dsRNA against the *Djatg8* genes after inducing. All RNAi-experiments in this study involved six feedings over 18 days (Fig. 5A) and amputations were carried out 24 h after final feeding (Fig. 5B). The regeneration pieces (head and tail pieces) were incubated in the autoclaved tap water and imaged for 14 days following injury. The effectiveness of RNAi was confirmed by RT-PCR and WISH techniques. The following sequence-specific primers were used:

Djatg8-1 RNAiF: 5′-TCTAGAGTAAAGTCTAAAATGAAGTGGCAG-3′,

Djatg8-1 RNAiR: 5′-GGTACCTCACCATTAACACGAATACGAG-3′,

Djatg8-2 RNAiF: 5′-TCTAGATTGGGTTAAATTAGTTAAGTCAAGT-3′,

Djatg8-2 RNAiR: 5′-GGTACCATTATGAAAAGTTGCCAAAGG-3′,

Djatg8-3 RNAiF: 5′-TCTAGAAATCAAATCCACTGTCATTCAT-3′ and

Djatg8-3 RNAiR: 5′-AAGCTTCAGGTCCGACGGGACAAGGTA-3′.

### Whole-mount immunostaining

Worms were treated with 2% HCl and fixed in paraformaldehyde with phosphate buffered saline (PBS) at 4°C for 4 h (Dong et al., 2019). Then, planarians were dehydrated with 100% methanol and rehydrated with 70%, 50%, 30%, 0% methanol. Subsequently, samples were labeled with anti-phospho Histone H3 (merck KGaA, Darmstadt Germany, no. 06-57) as a mitosis marker (González-Estévez et al., 2007a).

### qPCR

qPCR was carried out as described previously (Dong et al., 2017). The following sequence-specific primers were used:

Djatg8-1qPCRF: 5′-AAGCCCCTAAAGCCAGAGTTC-3′,

Djatg8-1 qPCRR: 5′-TAAATCCTCTTCGTGATGCTCTTC-3′,

Djatg8-2 qPCRF: 5′-CCTCAAGCAAGTATGACGATGG-3′,

Djatg8-2 qPCRR: 5′-AGAATTATGAAAAGTTGCCAAAGG-3′,

Djatg8-3 qPCRF: 5′-GCAGCAAGTTCAAGGACGAG-3′,

Djatg8-3 qPCRR: 5′-GTCCGACGGGACAAGGTAC-3′,

Djβ-actin qPCRF: 5′-ACACCGTACCAATCTATG-3′ and

Djβ-actin qPCRR: 5′-GTGAAACTGTAACCTCGT-3′.

### Statistical analysis

Statistical analyses of gene expression was carried out using SPSS 13.0 software via one-way analysis of variance (ANOVA) (Dong et al., 2017). *P*<0.05 was considered significant and *P*<0.01 was considered extremely significant.

## Supplementary Material

Supplementary information
