# Supplementary material for: Autophagy-related Djatg8 is required for remodeling in planarian Dugesia japonica
Source: Biol Open. 2019 Dec 3;8(12):bio045013. doi: 10.1242/bio.045013 (PMC6918785; doi:10.1242/bio.045013)
Supplement: Supplementary information [file biolopen-8-045013-s1.pdf]

Supplementary materials

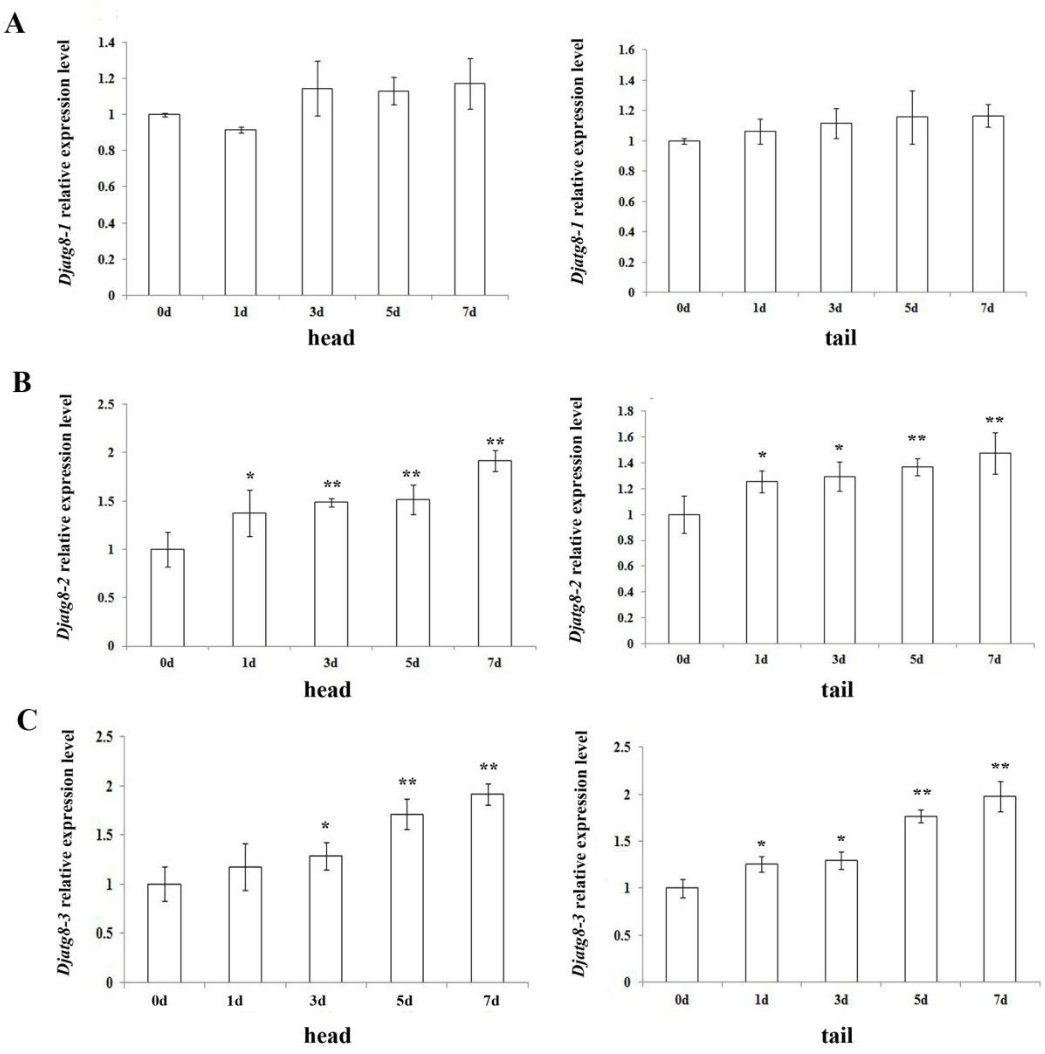

Fig. S1. The relative expression level of *DjAtg8s* in regenerating planarians (n = 10 for each treatment). (A) Quantitative PCR (qPCR) showing the relative expression level of *DjAtg8-1* in regenerating planarians; (B) Quantitative PCR (qPCR) showing the relative expression level of *DjAtg8-2* in regenerating planarians; (C) Qantitative RT-PCR (qPCR) showing the relative expression level of *DjAtg8-3* in regenerating planarians. Asterisks indicate statistical significance (*P < 0.05; **P < 0.01).

Biology Open • Supplementary information

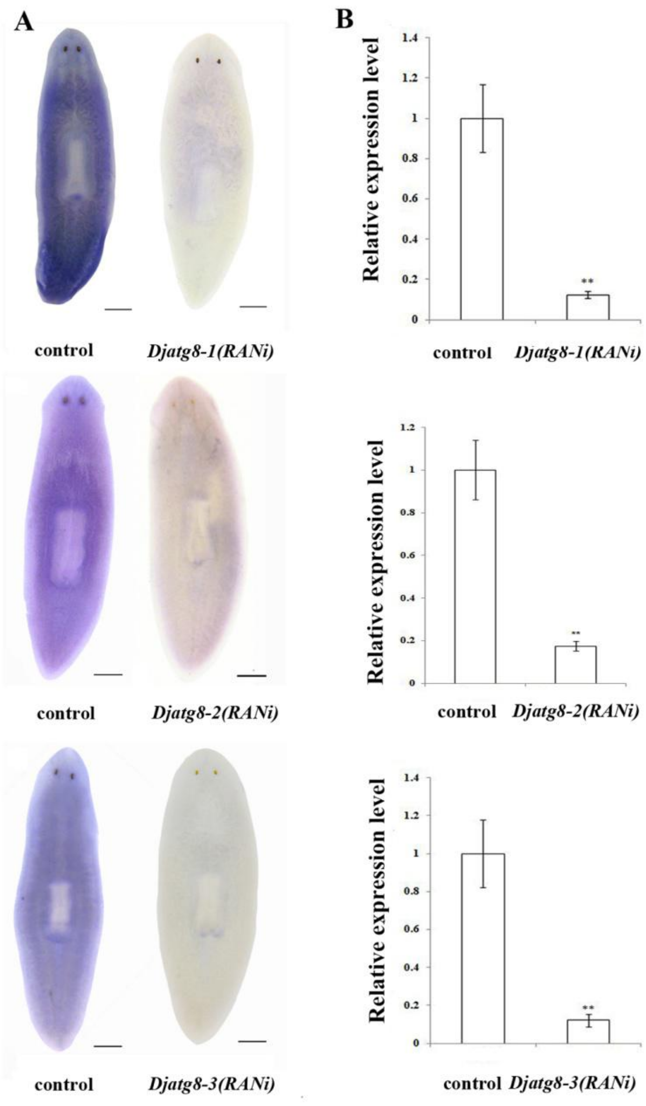

Fig.S2. The effective of *Djatg8s* RNAi. (A) WISH in RNAi planarians (n = 12 for each treatment); (B) qPCR was used to measure mRNA levels of *Djatg8s* (RNAi) (n = 10 animals for each treatment). Asterisks indicate statistical significance (*P < 0.05; **P < 0.01). Samples were collected at 8th d after feeding.

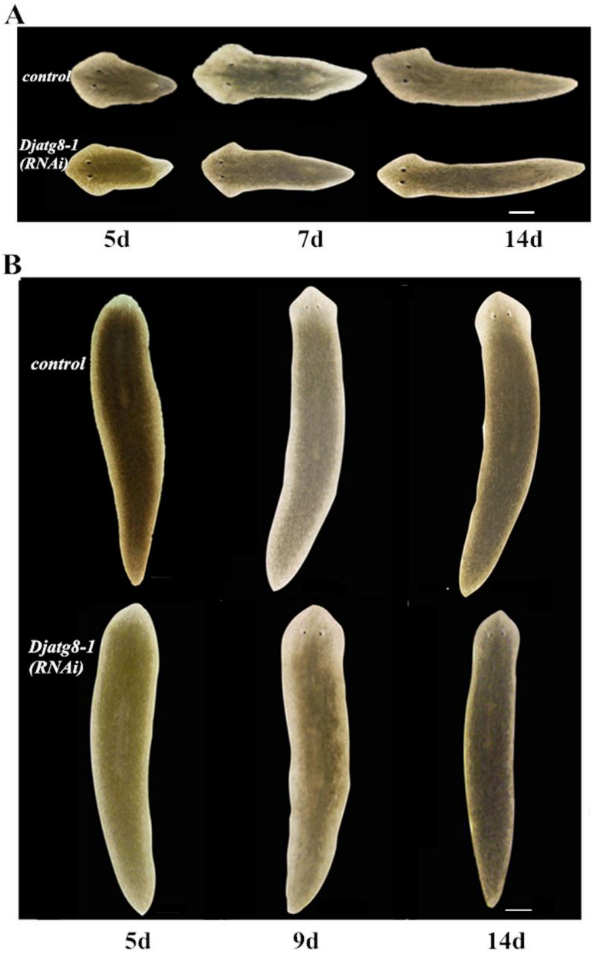

Fig.S3. The planarian phenotypes after *Djatg8-1* RNAi (n = 20 animals). (A) Head blastemas at 5, 7 and 14 days of regeneration, respectively; (B) Tail blastemas at 5, 9 and 14days of regeneration, respectively. Scale bar, 500 µm.

Biology Open • Supplementary information
